# Psychological transdiagnostic factors and migraine characteristics as predictors of migraine-related disability

**DOI:** 10.1186/s10194-025-02101-4

**Published:** 2025-07-23

**Authors:** Janosch Fox, Charly Gaul, Julia Ohse, Nicolina Peperkorn, Joshua Krutzki, Youssef Shiban

**Affiliations:** 1 Department of Psychology, PFH Goettingen, Goettingen, Germany; 2 Department of Psychiatry and Psychotherapy, University Medical Center Goettingen, Goettingen, Germany; 3 Medical Faculty, University of Duisburg-Essen, Essen, Germany; 4 Headache Center Frankfurt, Frankfurt a. M., Germany

**Keywords:** Migraine, Fear avoidance model, Disability, Predictors, Psychological, Biopsychosocial, Pain catastrophizing, Fear of attacks

## Abstract

**Background:**

Migraine is a prevalent primary headache disorder that significantly impairs daily life. Research on factors contributing to migraine-related disability remains limited, particularly from a biopsychosocial perspective. This study investigated whether transdiagnostic psychological factors, as proposed by the Fear Avoidance Model (FAM), contribute to migraine-related disability beyond migraine symptoms.

**Methods:**

In this cross-sectional analysis of selected baseline data from an ongoing randomized controlled trial, data from *N* = 158 individuals with migraine reporting ≥ 4 migraine days per month were examined. Data was collected through an online survey, including sociodemographic and clinical characteristics as well as responses to standardized questionnaires (DASS, PCS, FAMI, HIT-6). A hierarchical multiple linear regression analysis was conducted, including independent variables in two blocks: (1) sociodemographic factors and migraine symptoms, and (2) FAM factors (pain catastrophizing, fear of attacks, depressiveness). Disability, the dependent variable, was assessed using the HIT-6 questionnaire. Additionally, mediation analyses were conducted to investigate the potential mediating role of pain catastrophizing in the relationship between pain intensity or attack frequency and disability.

**Results:**

A total of *N* = 158 participants were included in the analysis. Hierarchical regression analysis showed that sociodemographic and migraine symptoms accounted for 49% of the variance in disability (*R²*_*adj*_ = 0.49, *p* < 0.001). The inclusion of FAM factors significantly increased the explained variance to 62% (*R²*_*adj*_ = 0.62, *p* < 0.001; *ΔR²*_*adj*_*=* 0.13, *p* < 0.001), indicative of a high goodness-of-fit. Independent predictors included gender *(ß* = -0.15, *p* = 0.007), age *(ß* = 0.11, *p* = 0.029), maximum pain intensity *(ß* = 0.46, *p* < 0.001), pain catastrophizing *(ß* = 0.26, *p* < 0.001), and depressiveness *(ß* = 0.13, *p* = 0.047). Mediation analyses revealed that pain catastrophizing (*ß* = 0.35, *p* < 0.001) mediated the relationship between pain intensity (*ß* = 0.47, *p* < 0.001) and disability (*R²* = 0.62, *p* < 0.001), whereas no mediation effect was observed for attack frequency as independent variable (X→Y: *ß* = -0.05, *p* = 0.44; X→ M: *ß* = -0.07, *p* = 0.26; M → Y: *ß* = 0.51, *p* < 0.001).

**Conclusion:**

The findings underscore the significant role of transdiagnostic psychological factors in migraine-related disability beyond migraine and sociodemographic characteristics. Pain catastrophizing emerged as an important mediator between pain experience and disability, which is in line with the assumptions of the FAM.

**Trial registration:**

German Clinical Trials Register (DRKS), DRKS-ID: DRKS00033893.

## Introduction

### Migraine

Migraine is a prevalent and disabling neurological disorder, affecting approximately 14% of the global population [1]. It is characterized by recurrent episodes of moderate to severe headache, often accompanied by nausea, photophobia, and phonophobia [2]. While pharmacological treatments can alleviate symptoms, migraine remains a primary condition that often persists over the lifespan and has a substantial impact on daily life [3]. Migraine affects multiple functional domains, including occupational, academic, social, and family responsibilities, making it the second leading cause of years lived with disability (YLD) worldwide [1]. Recent work has emphasized the multifactorial nature of migraine, involving not only biological mechanisms such as altered pain processing and cortical hyperexcitability, but also psychological and social influences, which may contribute to disease progression and disability [4].

### Assessing the burden of migraine

Research on migraine treatments has traditionally prioritized attack frequency as the primary outcome measure. However, focusing on attack frequency alone is insufficient to fully capture the impact of migraine on daily life, which also include ictal and interictal disability [5]. As a result, the incorporation of disability and other Patient-Reported Outcome Measures (PROMs) has gained increasing importance [6–9]. For instance, the International Headache Society defines treatment response in clinical trials primarily as a ≥ 50% reduction in attack frequency, while significant improvements in validated migraine-specific patient-reported outcome measures (PROMs) are also recognized as acceptable indicators of clinical benefit [10–12]. Moreover, recent findings suggest that higher disability is associated with increased direct and indirect costs, independent of headache frequency [13].

### Migraine-related disability

Migraine-related disability has emerged as an important and independent outcome with both clinical and scientific relevance. Following the International Classification of Functioning, Disability and Health (ICF) framework in accordance with the biopsychosocial model of the World Health Organization, disability is understood as the outcome of interactions between health conditions and contextual factors, including environmental and personal influences [14]. As suggested by Steiner et al. [8], migraine-related disability refers to physical, cognitive, and/or mental incapacities imposed by migraine, which may manifest either during or between migraine attacks [5].

In contrast to expectations, the association between attack frequency and disability ranges from none to only moderate [15–17]. This suggests that higher attack rates do not necessarily correspond to greater disability. Conversely, individuals with infrequent attacks may nonetheless report high levels of disability. It has also been shown that the relationship between attack frequency and quality of life remains linear up to approximately 13 headache days per month, after which it significantly decreases [18], suggesting a different significance of attack frequency in episodic versus chronic migraine. This dissociation underscores the multifactorial nature of migraine-related disability, revealing that the contributing factors and underlying mechanisms remain poorly understood.

A growing body of research highlights the importance of cognitive-affective and behavioral responses to pain as contributors to migraine-related disability. Psychological factors, such as pain catastrophizing, fear of attacks, and depression have been implicated in the exacerbation of disability (e.g., see [3, 19–22]), emphasizing the need for a biopsychosocial approach.

### Fear avoidance model (FAM)

The FAM [23–25] offers a promising theoretical framework for understanding how psychological factors contribute to disability within a biopsychosocial perspective on illness. According to the FAM, individuals with dysfunctional pain processing are more likely to develop fear of pain, pain hypervigilance, avoidance behaviors, and increased depressive symptoms, all of which reinforce disability. Pain catastrophizing, a maladaptive cognitive response to pain, is considered a key factor in this process.

The FAM was originally developed in the context of chronic back pain. Unlike musculoskeletal pain, migraine is characterized by episodic and often unpredictable attacks, which can lead to different patterns of fear-avoidance behavior. While fear of pain in musculoskeletal conditions typically results in the avoidance of physical activity, fear-avoidance behavior in migraine encompasses a broader range of potential triggers [26]. Emerging empirical evidence suggests that maladaptive cognitive-affective factors as postulated by the FAM (pain catastrophizing, fear of attacks, and depressiveness) are associated with higher levels of disability in various pain conditions, including migraine [22, 27, 28], suggesting a transdiagnostic role for this framework. However, further investigation is needed into the applicability of the FAM framework to migraine.

### Research gap

Despite the significant impact of migraine on daily life, no comprehensive theoretical framework currently exists that adequately explains the mechanisms underlying disability. Although current research provides valuable insights into individual contributing factors, it remains unclear how these factors collectively lead to disability. Few studies have investigated the contributing factors and underlying mechanisms of disability from a biopsychosocial perspective. This has left the complex interplay of biological and psychological factors in this context largely unexplored.

### Aim of the present study

Based on a theory-driven approach, this study examines whether psychological factors postulated by the FAM are related to migraine-related ictal disability. It is hypothesized that pain catastrophizing, fear of attacks, and depressiveness are independent predictors of ictal disability beyond migraine symptoms. Furthermore, it is hypothesized that pain catastrophizing acts as a mediator between pain experience and ictal disability. By integrating a biopsychosocial perspective, this study aims to explore potential mechanisms underlying migraine-related disability, with implications for both research and clinical practice.

## Methods

This study presents an analysis of data from an ongoing study (German Register of Clinical Trials, ID: DRKS00033893), focusing on sociodemographic and clinical characteristics at baseline as predictors of disability in individuals with migraine. Data collection for this study took place between April 2024 and February 2025 via an online survey using the LimeSurvey platform (LimeSurvey GmbH. LimeSurvey: An Open Source survey tool. http://www.limesurvey.org).

To minimize potential sources of bias, standardized recruitment and assessment procedures were used, along with predefined eligibility criteria and validated self-report instruments. All procedures and reporting follow the recommendations from the Strengthening the Reporting of Observational Studies in Epidemiology (STROBE) statement [29].

### Study population

Participants with a reported physician diagnosed migraine and an average of at least four attacks per month were eligible to participate. The diagnosis was validated using a questionnaire based on the International Classification of Headache Disorders, 3rd edition (ICHD-3) criteria [2]. Inclusion criteria were: (1) headache attacks lasting 4–72 h if untreated, (2) at least two characteristics of the following - unilateral localization, pulsating quality, moderate or severe intensity, aggravation by or causing avoidance of routine physical activity - and (3) at least one accompanying symptom (nausea and/or vomiting, photophobia and phonophobia). Exclusion criteria included withdrawal of informed consent, reported current drug or alcohol misuse, uncorrected visual or hearing impairments, psychotic symptoms, or cognitive impairments that could negatively affect data acquisition (e.g., dementia).

### Recruitment

Participants were recruited online via the official websites of the Migraine Patients Organization Germany (Migräne Liga e.V.), the German Migraine and Headache Society (DMKG e.V.), and the Headache Center Frankfurt. Additionally, participants were made aware of the study via printed notices in clinics and medical practices. Furthermore, participants were recruited through targeted outreach on the social media platforms Instagram and Facebook.

### Ethics

Prior to participation, all respondents received a written overview detailing the study’s objectives, the intervention, and the assessment procedures. Additionally, participants were informed about data collection, storage, and protection. Ethical approval for the study protocol was granted by the Ethics Committee of the PFH Göttingen (reference number: 251981/4) prior to the commencement of data collection on December 20, 2023. Furthermore, participants were informed of their rights to access, amend, delete, or restrict the use of their personal data. No financial incentives were provided for participating in the study. In accordance with data protection regulations, the data from this study will only be shared with qualified researchers upon reasonable request and in fully anonymized form following completion of the study.

### Power analysis

A priori power analysis was conducted for the hierarchical regression analysis (Model 1: nine predictors, Model 2: three additional predictors). Based on McCracken et al. [21], a large effect size (*f²* = 0.35) was expected for Model 1. For Model 2, a moderate effect size for the additional explained variance was anticipated, based on the meta-analysis by Rogers and Farris [28]. The power analysis resulted in a sample size of *N* = 132, with a significance level of α = 0.05 and a test power of 0.80. All power calculations were performed using G*Power version 3.1.9.7 [30].

### Measures

For the analysis, sociodemographic and clinical characteristics were collected. Sociodemographic variables included age and gender, with gender being reported as male, female, or diverse. Migraine characteristics encompassed self-reported attack frequency (number of migraine attacks during the past four weeks and average number of migraine days per month over the past 3 months according to ICHD-3 criteria [2]), course of disease (episodic vs. chronic migraine), pain intensity (mean and maximum pain intensity rated on a Visual Analog Scale [VAS] from 0 to 10 over the past four weeks), and the presence of accompanying symptoms, such as aura, nausea, and photophobia/phonophobia. In addition, screening of medication-overuse headache (MOH) was conducted by inquiring whether participants had used pain medication for headache on 10 or more days in each of the past three months. Episodic migraine (EM) was characterized as < 15 migraine days per month, while chronic migraine (CM) was characterized as ≥ 15 migraine days per month. Furthermore, psychological and transdiagnostic factors based on the FAM were assessed using standardized self-report measures. Migraine-related disability was assessed using the Headache Impact Test (HIT-6).

#### Depression, anxiety and stress scales (DASS)

The German version of the Depression, Anxiety and Stress Scales (DASS) [31–33] comprises three self-report scales designed to assess psychological distress. The subscales are depression, anxiety, and stress. Each consists of seven items rated on a 4-point Likert scale ranging from 0 (“did not apply to me at all”) to 3 (“applied to me very much”). Scores for each subscale are summed. Higher scores indicate greater symptom severity, with suggested cut-off scores of ten for depression, six for anxiety, and ten for stress [33].

#### Pain catastrophizing scale (PCS)

The Pain Catastrophizing Scale (PCS) [34] is a widely used 13 item instrument for assessing dysfunctional cognitive and affective responses to pain *on* three subdomains: rumination, magnification, and helplessness. Patients rate each item on a 5-point Likert scale ranging from 0 (“not at all”) to 4 (“all the time”), yielding a total score between 0 and 52, with higher scores indicating greater levels of pain catastrophizing. A total score of ≥ 30 is considered pathological, as this threshold corresponds to the 75th percentile among individuals with chronic pain [35].

#### Fear of attacks in migraine inventory (FAMI)

The Fear of Attacks in Migraine Inventory (FAMI) [36] is a recently developed self-report instrument designed to assess attack-related fear in individuals with migraine. containing the subscalesfear of negative consequences, attention and anticipation, and fear-avoidance [36]. The FAMI comprises a total of 29 items, rated on a 5-point Likert scale ranging from 1 (“strongly disagree”) to 5 (“strongly agree”). A cutoff score of ≥ 116 has been proposed [37]. Initial psychometric evaluation [36] supports the FAMI’s reliability and validity. Confirmatory factor analysis indicated acceptable to good model fit. Reliability was good to excellent across subscales, and convergent validity was confirmed through correlational analyses.

#### Headache impact test (HIT-6)

The six-item Headache Impact Test (HIT-6) is a screening tool for assessing the impact of headaches in both clinical practice and research [38], by evaluating their impact on social functioning, role functioning, vitality, cognitive functioning, and psychological distress. For each of the six items, five answering options are provided (6 = “Never”; 8 = “Rarely”; 10 = “Sometimes”; 11 = “Very Often”; 13 = “Always”). The final score can be obtained by summing the six item responses, yielding a range of 36 to 78, with a higher score indicating a greater impact [39]. Scoring conventions range from “little or no impact” (≤ 49), over “some impact” (50–55) and “substantial impact”(56–59), up to “severe impact” (60–78) [39].

#### Pain intensity

Pain intensity was assessed by evaluating participants’ average and maximum pain intensity over the past four weeks. For this, a Visual Analog Scale (VAS) was used, as this is a common tool in migraine research for measuring participants’ pain [40]. Participants rated both their average and maximum pain intensity on a horizontal scale ranging from 0 (“no pain”) to 10 (“unbearable pain”), with higher scores indicating greater perceived pain severity.

### Statistical analysis

Statistical analyses were performed using the Statistical Package for the Social Sciences (SPSS Statistics; IBM, version 28, Germany). To ensure the integrity and reliability of the analysis, all incomplete data sets were excluded. Subsequently, socio-demographic and clinical variables were screened for potential outliers to minimize measurement errors. Although some outliers were detected, all values were deemed plausible, and therefore, no data was removed. The distribution of continuous and discrete variables was visually assessed using histograms and Q-Q plots.

Measures of central tendency (mean or median), measures of variability (standard deviation (SD) or interquartile range (IQR)) and frequency distributions (absolute (N or n) and relative frequency (%)) were used to summarize the characteristics of the study population and outcomes. To examine differences between participants with pathological vs. non-pathological levels of pain catastrophizing, group comparisons were conducted using independent *t*-tests or Mann-Whitney *U-*tests, depending on the data distribution. Reporting and interpretation of effect sizes follow the conventions set by Cohen [41]. Specifically, a correlation coefficient (*r*) of 0.1 is considered a weak correlation, 0.3 a moderate correlation, and 0.5 or higher a strong correlation. For standardized mean differences, Cohen’s *d* values of 0.2, 0.5, and 0.8 indicate small, medium, and large effects, respectively. Derived from Cohen’s [41] guidelines for *f*^*2*^, the corresponding *R*^*2*^ values for effect sizes in regression analysis are considered as follows: an *R*^*2*^ of 0.02 is considered a small effect, 0.13 a medium effect, and 0.26 a large effect.

To test the directional hypothesis, a hierarchical multiple linear regression was conducted. In Model 1, demographic and clinical variables were included as predictors: gender, age, attack frequency, migraine type (episodic migraine (EM) vs. chronic migraine (CM)), aura symptoms, nausea, photophobia/phonophobia, maximum pain intensity, and mean pain intensity. In Model 2, three psychological factors derived from the theoretical framework of the FAM were added to examine their incremental predictive value: pain catastrophizing, fear of attacks, and depressiveness. The change in explained variance (*ΔR²)* between the models was used to determine whether the additional predictors significantly improved the model’s explanatory power.

Furthermore, two mediation analyses were conducted to investigate whether pain catastrophizing (mediator, M) mediated the relationship between pain experience (independent variable, X) and disability (dependent variable, Y), as postulated by the FAM. In mediation model 1, pain intensity was used as the independent variable, whereas in mediation model 2, attack frequency was used as the independent variable. To control for potential confounders, all significant predictors from the hierarchical regression analysis were included as covariates. Mediation analyses were performed using the PROCESS macro (v2.4) for SPSS [42]. Bootstrapping with 5,000 iterations was applied to ensure robust parameter estimation. Bias-corrected 95% confidence intervals (CIs) were used to assess the statistical significance of indirect effects.

A *p*-value of less than 0.05 is considered statistically significant for all analyses.

## Results

### Study population

**Fig. 1:**
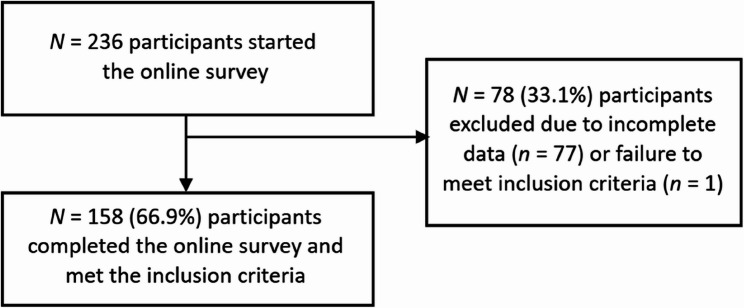
Flow chart of attrition

Out of 236 initiated survey attempts, 78 cases (33.1%) were excluded due to incomplete data (*n* = 77) or failure to meet inclusion criteria (*n* = 1). One case, identifying as diverse gender, was excluded because individuals with diverse gender identities were underrepresented in the study sample. A final sample of *N* = 158 complete datasets was used for the analysis. Data inspection did not reveal implausible values. Figure 1 depicts the study’s attrition process.

### Sociodemographic and clinical characteristics

Table 1 presents the sociodemographic and migraine characteristics of the study sample. The majority of participants identified as female (84.8%), while 14.6% reported a male gender and 0.6% identified as diverse. The mean age of the sample was *M* = 36.9 years (*SD* = 12.5), ranging from 18 to 71 years. A total of 27.8% of participants (*n* = 44) reported a diagnosed psychiatric disorder (e.g., depression, anxiety).

**Table 1:** Sociodemographic and migraine characteristics of the sample, *N = *158

Characteristic		Value
Age, mean ± SD [range]	years	36.92 ± 12.47 [18–71]
Gender, *n* (%)	female	134 (84.8)
	male	23 (14.6)
	diverse	01 (0.6)
Migraine type, *n* (%)	episodic migraine	138 (87.3)
	chronic migraine	20 (12.7)
Aura		67 (42.4)
Nausea, *n* (%)		106 (67.1)
Photophobia/phonophobia, *n* (%)		141 (89.2)
Attack frequency, Mdn (IQR) [range]	average number of migraine days (past 3 months)	6.0 (4.75-10.0) [4–31]
	number of migraine attacks (past 4 weeks)	6.0 (4.0–09.0) [01–28]
Screening of medicaton-overuse headache, *n* (%)	positive screening	8 (5.1)

*n* number valid cases, *SD* standard deviation, *Mdn* median, *IQR* interquartile range

The median number of migraine days in both the past three months and the past four weeks was 6.0 (*IQR* = 4.75–10.0; *IQR* = 4.0–9.0). A total of 12.7% of participants reported experiencing an average of 15 or more migraine days per month over the past three months, which is indicative of chronic migraine. Screening for medication-overuse headache (MOH) revealed possible MOH in 8 participants (5.1%).

Table 2 presents the clinical characteristics of the study sample. The mean HIT-6 disability score was *M* = 65.2 (*SD* = 5.8). The majority of participants (90.5%) reported severe migraine-related disability, while 4.4% experienced a substantial impact. Only a small proportion reported moderate (2.3%) or little to no disability (1.9%). Regarding psychological distress, 32.3% of participants exhibited pathological screening scores on the DASS depression subscale (score ≥ 10), 29.8% on the DASS anxiety subscale (score ≥ 6), and 44.3% on the DASS stress subscale (score ≥ 10). A pathological pain catastrophizing score (> 30) was observed in 35.4% of participants, while 15.2% reported a pathological fear of attacks score (≥ 116).

**Table 2:** Clinical characteristics of the sample, *N* = 158

Characteristic	Value	Value range
HIT-6, mean ± SD [range]	65.21 ± 5.86 [36–78]	36–78
Maximum pain intensity (VAS), mean ± SD [range]	7.43 ± 1.72 [1–10]	0–10
Mean pain intensity (VAS), mean ± SD [range]	5.61 ± 1.62 [1–9]	0–10
DASS Depression, mean ± SD [range]	7.28 ± 5.47 [0–21]	0–21
DASS Anxiety, mean ± SD [range]	4.39 ± 4.09 [0–19]	0–21
DASS Stress, mean ± SD [range]	8.89 ± 4.53 [0–21]	0–21
PCS, mean ± SD [range]	25.23 ± 11.48 [0–47]	0–52
PCS Subscale Rumination, mean ± SD [range]	9.25 ± 4.01 [0–16]	0–16
PCS subscale Magnification, mean ± SD [range]	4.71 ± 2.86 [0–12]	0–12
PCS Subscale Helplessness, mean ± SD [range]	11.27 ± 5.96 [0–24]	0–24
FAMI, mean ± SD [range]	94.63 ± 21.62 [29–140]	29–140
FAMI Subscale Fear of negative consequences, mean ± SD [range]	31.15 ± 9.1 [9–45]	9–45
FAMI Subscale Attention and anticipation, mean ± SD [range]	41.07 ± 9.77 [13–63]	13–65
FAMI Subscale Fear-Avoidance, mean ± SD [range]	22.41 ± 5.39 [7–34]	7–35

*VAS* Visual Analog Scale, *HIT-6* Headache Impact Test, *DASS* Depression Anxiety and Stress Scales, *PCS *Pain Catastrophizing Scale, *FAMI* Fear of Attacks in Migraine Inventory, *SD* standard deviation, *IQR* interquartile range

### Zero-order correlations

Table 3. presents zero-order correlations between disability, migraine characteristics, and psychological variables, providing a preliminary overview of their bivariate relationships. Disability showed significant correlations with nearly all examined variables, except for attack frequency, course of disease, and aura symptoms. The strongest associations were observed for mean pain intensity (*r* = 0.53, *p* < 0.001) and maximum pain intensity (*r* = 0.65, *p* < 0.001), followed by weaker correlations with photophobia/phonophobia (*r* = 0.26, *p* < 0.001) and nausea (*r* = 0.18, *p* = 0.023). Both anxiety and stress exhibited moderate correlations with disability (*r* = 0.42, *p* < 0.001; *r* = 0.48, *p* < 0.001) and strong, significant correlations (*r* ≥ 0.50, *p* < 0.001) with the FAM-factors except for fear of attacks, which only demonstrated a moderate correlation (*r* = 0.44, *p* < 0.001).

**Table 3:**
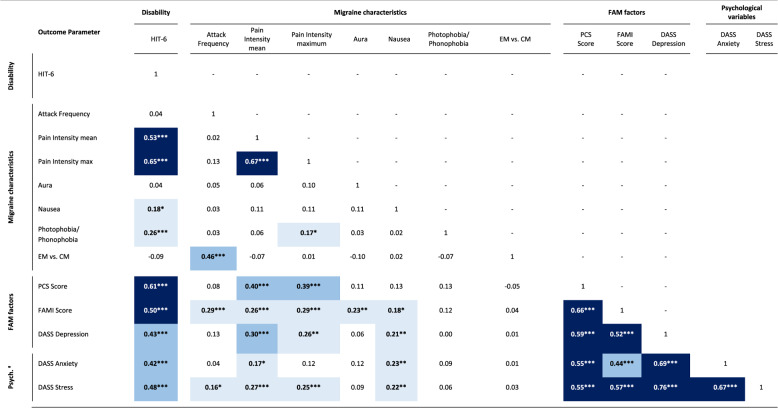
Zero-order correlations between disability, migraine characteristics, and psychological outcome parameters, *N* = 158

*a Psych* Psychological variables, *FAM* Fear Avoidance Model, *HIT-6* Headache Impact Test, *PCS* Pain Catastrophizing Scale, *DASS* Depression Anxiety and Stress Scales, *FAMI* Fear of Attacks in Migraine Inventory. r, Pearson's rho, Spearman's rho used for correlations with attack frequency, aura, nausea, photophobia, EM vs. CM; p, p-value; Significant correlations are marked in bold (two-tailed); light blue, significant weak correlation (r ≥ 0.10); blue, significant moderate correlation (r ≥ 0.30); dark blue, significant strong correlation (r ≥ 0.50).

FAM-related psychological variables were also significantly associated with disability. Pain catastrophizing (*r* = 0.61, *p* < 0.001) and fear of attacks (*r* = 0.50, *p* < 0.001) showed strong associations, while depressiveness demonstrated a moderate correlation (*r* = 0.43, *p* < 0.001). Additionally, FAM-related variables demonstrated strong intercorrelations (*r* > 0.50). Pain catastrophizing was significantly associated with both mean (*r* = 0.40, *p* < 0.001) and maximum pain intensity (*r* = 0.39, *p* < 0.001). Fear of attacks (*r* = 0.29, *p* < 0.001) and stress (*r* = 0.16, *p* = 0.041) were the only psychological variables significantly correlated with attack frequency, alongside the course of disease (*r* = 0.46, *p* < 0.001). Fear of attacks also showed small to moderate correlations with most migraine-related characteristics, with the exception of photophobia/phonophobia (*r* = 0.12, *p* = 0.133) and course of migraine (*r* = 0.04, *p* = 0.621).

### Prediction of pain-related disability

**Fig. 2:**
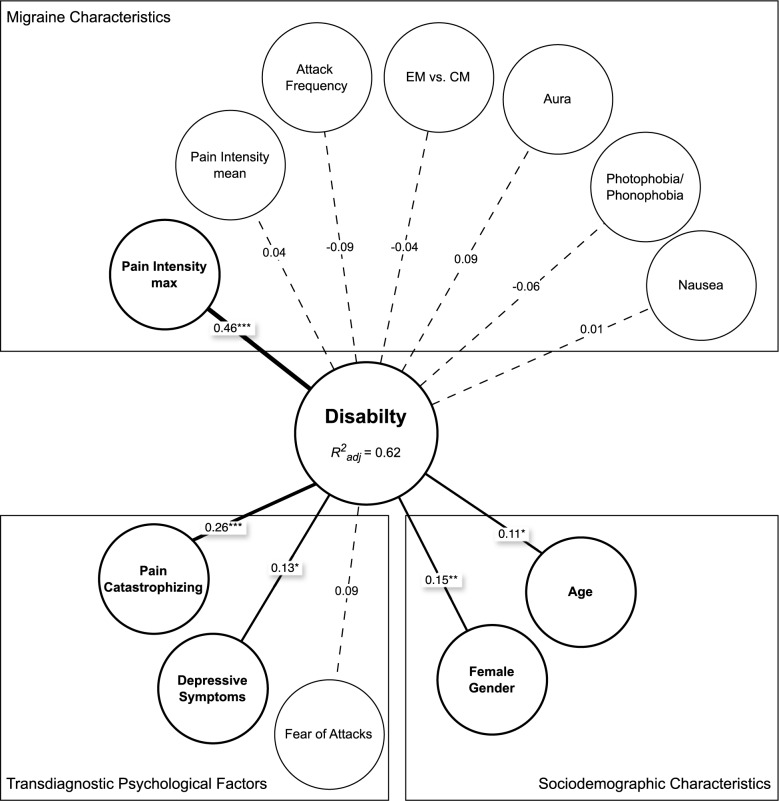
Predictors of Migraine-related Disability: Results of the Regression Analysis, *N* = 158. *R*^*2*^_*adj*_, adjusted coefficient of determination. Numbers on lines represent standardized regression coefficients. *, *p* < 0.05; **, *p* < 0.01; ***, *p* < 0.001

A hierarchical multiple linear regression analysis was conducted to examine the relationship between disability and the FAM factors, controlling for migraine symptoms. The results for this analysis are presented in Table 4 and illustrated in Fig. 2.

Model 1 included gender (female vs. male), age, attack frequency, course of disease (EM vs. CM), aura symptoms, nausea, photophobia/phonophobia, and both maximum and mean pain intensity as predictors. This model explained 49% of variance in disability (*R²*_*adj*_ = 0.49, *p* < 0.001), indicating a high goodness-of-fit. Among the included independent variables, gender (*ß* = −0.14, *p* = 0.02), aura symptoms (*ß* = 0.14, *p* = 0.02), and maximum pain intensity (*ß* = 0.55, *p* < 0.001) were found to be independent predictors of disability. The coefficients of the significant predictors indicate that female gender, the presence of aura symptoms, and higher pain intensity were associated with increased disability.

In Model 2, the inclusion of the FAM factors resulted in an increase in explained variance in disability. The change in *R²* was *ΔR²*_*adj*_*=* 0.13, which corresponds to a medium effect size. The final model explained a total of 62% in disability (*R²*_*adj*_ = 0.62, *p* < 0.001), demonstrating a high goodness-of-fit. Regression analysis identified gender *(ß* = −0.15, *p* = 0.007), age *(ß* = 0.11, *p* = 0.029), maximum pain intensity *(ß* = 0.46, *p* < 0.001), pain catastrophizing *(ß* = 0.26, *p* < 0.001), and depressiveness *(ß* = 0.13, *p* = 0.047) as significant independent predictors of disability. Aura symptoms did no longer reach statistical significance *(ß* = 0.09, *p* = 0.089). The mean disability score was 45.9. Female gender and higher age were both associated with greater disability. Each one-point increase in maximum pain intensity was associated with a 0.46-point increase in disability. For every one-point increase on the pain catastrophizing scale, disability increased by 0.26 points, while each one-point increase on the depressiveness scale was associated with a 0.13-point increase in disability.

**Table 4:** Results of the hierarchical multiple linear regression analyses, assessed with the HIT-6 as outcome, *N* = 158

Model	*R*	*R* ^2^	*R* ^2^_adj_	*p*	Change in *R*^2^		ß	B	SE_B_	95%-CI*	
					ΔR²_adj_	*p*				Lower bounds	Upper bounds
Model 1	0.72	0.52	0.49	**< 0.001**	-	-	-	-	-	-	-
Intercept				**< 0.001**			-	49.48	2.66	44.22	54.74
Gender				**0.020**			−0.14	−2.34	1.00	−4.31	−0.37
Age				0.118			0.09	0.04	0.03	−0.01	0.10
Attack Frequency				0.985			−0.00	−0.00	0.09	−0.17	0.17
EM vs. CM				0.159			−0.11	−1.88	1.33	−4.51	0.75
Aura symptoms				**0.020**			0.14	1.64	0.70	0.26	3.01
Nausea				0.465			−0.04	−0.53	0.72	−1.96	0.90
Photophobia/phonophobia				0.134			−0.09	−1.68	1.12	−3.89	0.52
Maximum Pain Intensity				**< 0.001**			0.55	1.88	0.28	1.33	2.43
Mean Pain Intensity				0.091			0.14	0.49	0.29	−0.08	1.05
Model 2	0.81	0.65	0.62	**< 0.001**	0.13	**< 0.001**	-	-	-	-	-
Intercept				**< 0.001**			-	45.90	2.58	40.80	50.99
Gender				**0.007**			−0.15	−2.40	0.87	−4.12	−0.68
Age				**0.029**			0.11	0.05	0.02	0.01	−0.10
Attack Frequency				0.214			−0.09	−0.10	0.08	−0.25	0.06
EM vs. CM				0.548			−0.04	−0.71	1.18	−3.04	1.62
Aura symptoms				0.089			0.09	1.06	0.62	−0.16	2.28
Nausea				0.849			0.01	0.12	0.63	−1.13	1.38
Photophobia/phonophobia				0.281			−0.056	−1.05	0.97	−3.00	0.87
Maximum Pain Intensity				**< 0.001**			0.462	1.585	0.25	1.10	2.07
Mean Pain Intensity				0.588			0.038	0.137	0.25	−0.36	0.64
Pain Catastrophizing				**< 0.001**			0.260	0.133	0.04	0.06	0.21
Fear of Attacks				0.245			0.086	0.023	0.02	−0.02	0.06
Depressiveness				**0.047**			0.131	0.139	0.07	0.00	0.28

Significant results in bold

*CI* Confidence interval, *EM* episodic migraine, *CM* chronic migraine

### Differences in clinical parameters due to pain catastrophizing

Clinical characteristics between participants with non-pathological and pathological pain catastrophizing scores were compared. The analysis revealed significant differences (*p* < 0.05) in disability, maximum and mean pain intensity, as well as in all psychological parameters (anxiety, stress, fear of attacks, depressiveness), with medium to large effect sizes. No significant differences (*p* > 0.05) were found regarding attack frequency, course of disease (EM vs. CM), or accompanying symptoms (aura symptoms, nausea, photophobia/phonophobia). The results for this analysis are presented in Table 5.

**Table 5:** Comparison of clinical characteristics between participants with non-pathological and pathological pain catastrophizing scores, *N* = 158

Parameter	PCS score ≤ 30 (*n* = 102)	PCS score > 30 (*n* = 56)	*p*-value	Effect size
Attack frequency (past 4 weeks), *Mdn* (*IQR*)	5.0 (4.0–9.0)	6.0 (3.0–9.0)	0.919	*r* = 0.01
Attack frequency (past 3 months), *Mdn* (*IQR*)	6.0 (4.0–10.0)	6.0 (5.0–10.0)	0.482	*r* = 0.06
Disability (HIT-6), *M* (*SD*)	63.15 (5.71)	68.96 (3.98)	**< 0.001**	*|d|****=*** 1.13
Maximum pain intensity (VAS), *M* (*SD*)	7.04 (1.86)	8.14 (1.14)	**< 0.001**	*|d|****=*** 0.67
Mean pain intensity (VAS), *M* (*SD*)	5.23 (1.53)	6.30 (1.57)	**< 0.001**	*|d|****=*** 0.70
DASS Depression, *M* (*SD*)	5.40 (4.32)	10.70 (5.73)	**< 0.001**	*|d|****=*** 1.01
DASS Anxiety, *M* (*SD*)	2.96 (2.82)	7.00 (4.72)	**< 0.001**	*|d|****=*** 1.12
DASS Stress, *M* (*SD*)	7.37 (3.65)	11.66 (4.68)	**< 0.001**	*|d|****=*** 1.06
FAMI Sum score, *M* (*SD*)	86.81 (20.49)	108.88 (15.60)	**< 0.001**	*|d|****=*** 1.17
FAMI Subscale Fear of negative consequences, *M* (*SD*)	28.38 (9.16)	36.20 (6.50)	**< 0.001**	*|d|****=*** 0.94
FAMI Subscale Attention and anticipation, *M* (*SD*)	37.58 (9.05)	47.43 (7.65)	**< 0.001**	*|d|****=*** 1.15
FAMI Subscale Fear-Avoidance, *M* (*SD*)	20.90 (5.26)	25.25 (4.40)	**< 0.001**	*|d|****=*** 0.88

Independent *t*-tests were conducted, except for attack frequency (Mann-Whitney *U* test). Effect sizes: Cohen’s *d* for t-tests, correlation coefficient (*r*) for Mann-Whitney *U* test. Two-tailed significance, significant results in bold

### Mediation analysis

Two mediation models were conducted to examine the relationship between pain experience and disability, with pain catastrophizing as mediator. A comprehensive table presenting all statistical values can be found in Tables 6 and 7 in the appendix.

**Fig. 3:**
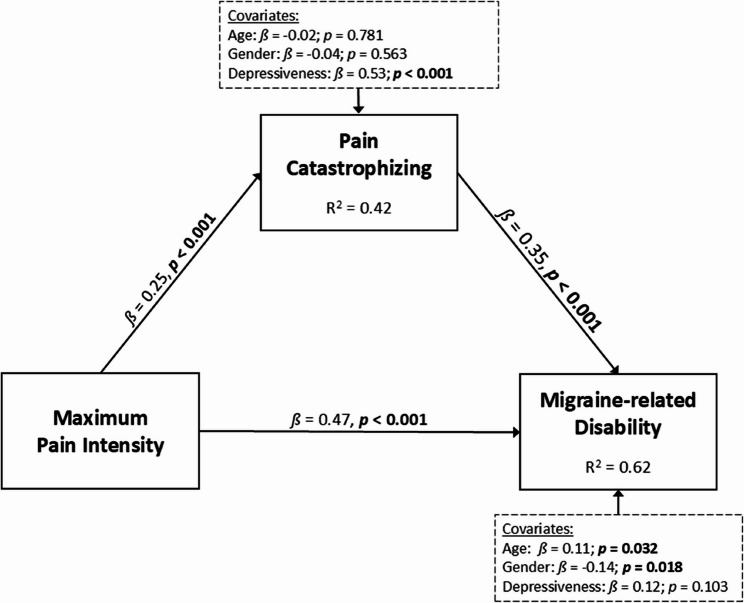
Mediation model of the relationship between pain experience and disability, with pain catastrophizing as the mediator. *ß*, standardized regression coefficient. Variables in dashed boxes represent controlled factors. Significant results in bold

The mediation model 1, with pain intensity as the independent variable, was significant, accounting for 62% of variance in disability (*R²* = 0.62, *p* < 0.001), indicating a large effect size. Pain catastrophizing (*ß* = 0.35, *p* < 0.001) was a significant mediator in the relationship between maximum pain intensity (*ß* = 0.47, *p* < 0.001) and disability. Among the controlled variables, gender and age were significantly associated with disability, with female gender and older age linked to higher disability levels (*ß* = −0.14, *p* = 0.018; *ß* = 0.11, *p* = 0.032). Depressive symptoms were significantly associated with pain catastrophizing (*ß* = 0.53, *p* < 0.001) but not with disability in this mediation model (*ß* = 0.12, *p* = 0.103). Furthermore, maximum pain intensity (*ß* =0.25, *p* < 0.001) was found to be a significant predictor of pain catastrophizing. Together, depressive symptoms and maximum pain intensity accounted for 42% of variance in pain catastrophizing (*R²* = 0.42, *p* < 0.001), indicating a large effect size. Figure 3 illustrates the results of mediation model 1. A full overview of all coefficients and model parameters is presented in Table 6 in (Appendix).

**Fig. 4:**
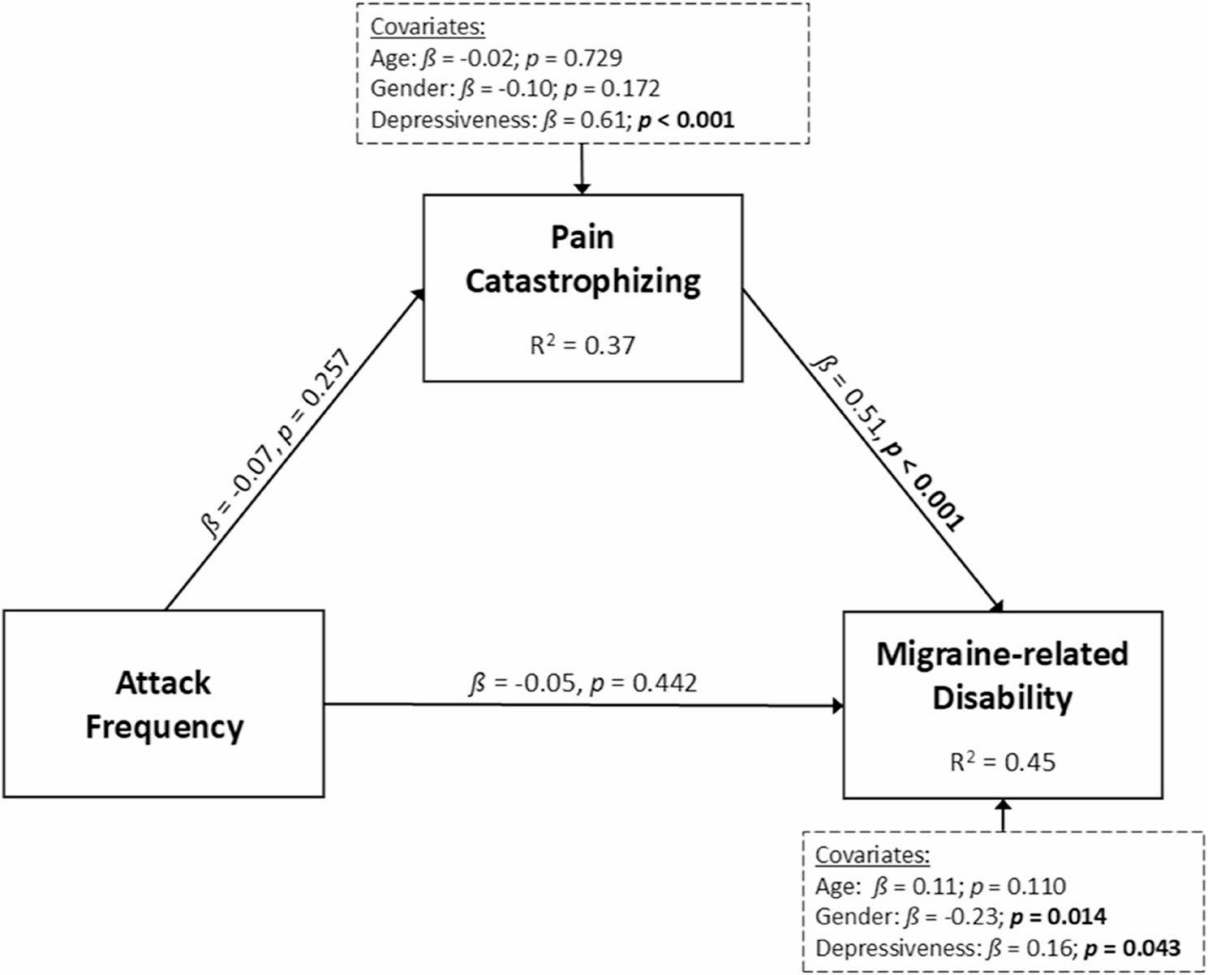
Mediation model of the relationship between attack frequency and disability, with pain catastrophizing as the mediator. *ß*, standardized regression coefficient. Variables in dashed boxes represent controlled factors. Significant results in bold

In mediation model 2, where attack frequency was the independent variable, neither the relationship between attack frequency (*ß* = −0.05, *p* = 0.442) and disability, nor the relationship between attack frequency (*ß* = −0.07, *p* = 0.257) and pain catastrophizing, was significant. The only significant relationship in this model was found between pain catastrophizing and disability (*ß* = 0.51, *p* < 0.001). Therefore, according to Zhao et al. [43], no mediation effect was found. The detailed results of mediation model 2 are summarized in Table 7 in (Appendix). Figure 4 illustrates the results of mediation model 2.

**Fig. 5:**
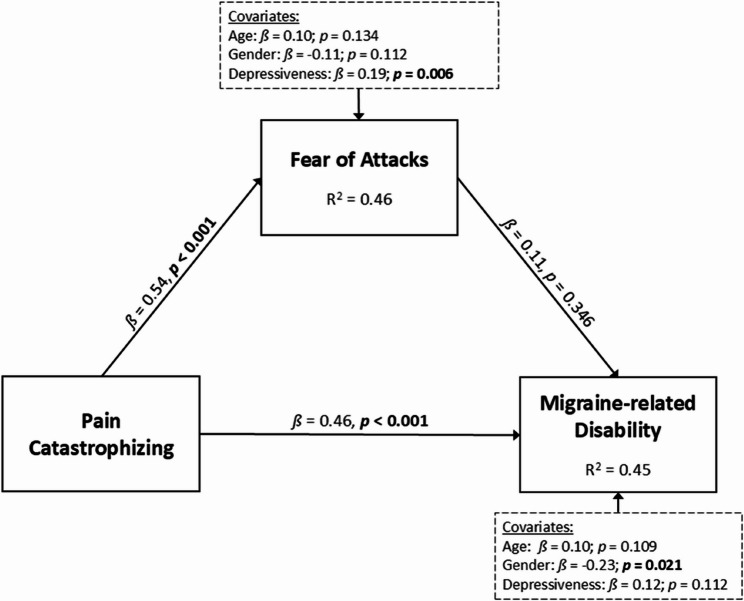
Mediation model of the relationship between pain catastrophizing and disability, with fear of attacks as the mediator. *ß*, standardized regression coefficient. Variables in dashed boxes represent controlled factors. Significant results in bold

Given that fear of attacks did not emerge as a significant predictor in the regression analysis, a third, exploratory mediation analysis was performed. This analysis examined the potential mediating role of fear of attacks in the association between pain catastrophizing and disability, while controlling for age, gender, and depressive symptoms. The model explained 45% of the variance in disability (R² = 0.45, *p* < 0.001). Pain catastrophizing was significantly associated with both fear of attacks (*ß* = 0.54, *p* < 0.001) and disability (*ß* = 0.46, *p* < 0.001). The indirect effect of pain catastrophizing on disability via fear of attacks was not statistically significant (*ß* = 0.07, *p* = 0.470). Among the covariates, female gender was significantly associated with disability (*ß* = −0.23, *p* = 0.021), whereas age and depressive symptoms were not significant predictors in this model. All statistical parameters (Table 8) and a figure (Fig. 5) for this mediation analysis are provided in the appendix.

## Discussion

Grounded in a biopsychosocial perspective, this study examined transdiagnostic psychological factors as predictors of migraine-related disability. To our knowledge, this is the first study to explicitly investigate predictors of disability in migraine using the FAM as a theoretical framework, thereby addressing a gap in migraine research. Employing a hierarchical multiple regression approach, it was found that sociodemographic and clinical migraine characteristics accounted for 49% of variance in disability. Including FAM factors significantly improved the model’s explanatory power, increasing the variance accounted for to 62%. Pain catastrophizing and depressive symptoms emerged as independent predictors of disability, whereas, contrary to expectations, fear of attacks did not. Notably, pain catastrophizing was identified as a strong mediator for the relationship between pain intensity and disability. Remarkably, frequency of migraine attacks and accompanying symptoms were not significantly associated with disability in this study.

### Findings in context with the FAM

The FAM posits that maladaptive cognitive-affective and behavioral responses to actual or anticipated pain play a central role in the development and maintenance of disability. Pain catastrophizing, fear of attacks, and depressive symptoms have been proposed as key psychological factors contributing to disability in individuals with pain conditions.

In line with the theoretical assumptions of the FAM, the present study identified pain catastrophizing and depressive symptoms as important predictors of disability. Although various migraine-related characteristics were also considered as potential predictors, only maximum pain intensity significantly predicted disability. This is at odds with previous studies, which found an association between greater frequency of migraine attacks and disability [44, 45]. One potential explanation for the lack of association between attack frequency and migraine-related disability observed in this study may lie in the conceptual distinction between ictal and interictal disability. In this study, ictal disability was assessed using the HIT-6 questionnaire. Recent evidence suggests that headache frequency seems to be strongly associated with interictal disability [46]., Furthermore, the assessment of attack frequency and disability was based on a self-report questionnaire rather than a diary method, which may have made it susceptible to recall bias [47]. Another potential explanation is the overrepresentation of participants severely affected by migraine (according to their HIT-6 scores) within this study. This restricted variability may have obscured a potential correlation. Furthermore, the results empirically support the notion that pain catastrophizing plays an important role in migraine-related disability, aligning with previous research findings [21, 22, 28, 48, 49]. Disability, pain intensity, and increased levels of depressive symptoms, stress, anxiety, and fear of attacks were all significantly higher in individuals with pathological pain catastrophizing scores compared to those with non-pathological scores. In contrast, frequency of attacks, course of disease, and accompanying symptoms were independent of pathological pain catastrophizing. Mediation analysis further revealed that pain catastrophizing is a crucial mediator in the relationship between pain intensity - but not attack frequency - and disability. This suggests that individuals who indicated higher levels of pain catastrophizing, i.e., those who tended to have dysfunctional cognitive pain processing, are more likely to experience a higher level of disability.

Taken together, these findings align with the assumptions of the FAM, which posits that higher levels of disability are not solely driven by the presence of pain itself but rather by the subjective pain experience and its maladaptive cognitive-affective processing.

### Fear of attacks and disability

While the results of the present study largely support the assumptions of the FAM, contrary to expectations, fear of attacks did not emerge as a significant predictor of disability. Although previous research has pointed to its role in pain-related disability [21, 36, 50], our study, which included several relevant predictors, did not support this finding. An exploratory post-hoc analysis demonstrated a strong association between pain catastrophizing and fear of attacks, but similarly did not identify fear of attacks as a mediator between pain catastrophizing and disability. Several explanations may account for this finding. Firstly, this discrepancy may suggest that fear of attacks affects disability only in specific subgroups of patients or that it operates through interaction effects. This warrants further longitudinal investigation, as such effects may not be detectable in a cross-sectional design. Secondly, fear of attacks may primarily reflect a psychophysiological response closely tied to the experience of previous attacks, rather than a distinct cognitive factor. This aligns with the dual-process theory, which suggests that implicit emotional responses (e.g., autonomic arousal) may operate independently from explicit, reflective emotional awareness. Supporting this, diary studies have shown increases in stress, negative affect, and psychological arousal before and during migraine episodes [51–53]. Zero-order correlations revealed that, apart from the course of disease, fear of attacks was the only variable significantly associated with attack frequency. Furthermore, its moderate correlations with aura symptoms, nausea, and pain intensity suggest that fear of attacks may be more closely tied to the clinical characteristics of migraine itself than to maladaptive cognitive-behavioral processes underlying disability. Thirdly, stigma-related expectations may constitute a separate fear pathway that contributes to disability. In this case, the fear would not be directed at the attack itself, but rather at the anticipated or actual negative social consequences resulting from the stigmatization associated with the attack. Studies have shown that a significant proportion of migraine patients experience stigmatization, which is linked to greater disability, higher interictal burden, and a reduced quality of life [54, 55].

### Predicting pain catastrophizing

A further key finding of this study is that maximum pain intensity and depressive symptoms accounted for 42% of the variance in pain catastrophizing. While depressive symptoms predicted pain catastrophizing, they were not directly linked to disability in this mediation model. The question of why some patients engage in pain catastrophizing while others do not remains a central, yet underexplored, issue in pain research. According to the FAM, both individual pain experiences and negative affectivity, alongside threatening illness information, are critical contributors. Further studies are needed to confirm these findings and clarify their implications for targeted interventions.

### Limitations

Several limitations should be considered when interpreting the results of this study. The cross-sectional design of the study limits the ability to draw causal conclusions. Longitudinal studies are essential to elucidate the temporal relationships between the FAM factors and disability. The primary study underlying this data analysis, designed as an RCT, aims to investigate the effects of pain catastrophizing, and the results of this ongoing trial are expected to provide further empirical insights to enhance the understanding of these interactions. Furthermore, the sample was predominantly female (84.8%). While this is consistent with migraine prevalence trends, it limits the generalizability of the findings, as females with migraine are more likely to experience a higher attack frequency, a longer duration of the attacks, greater disability, and increased rates of psychiatric comorbidities [56, 57]. Moreover, based on HIT-6 scores, more than 90% of participants reported severe migraine-related disability. This overrepresentation of severely affected individuals may have introduced a bias in the estimates, thereby limiting the generalizability of the findings to patients with lower levels of disability. However, investigating the mechanisms underlying disability remains particularly relevant in this population, as migraine-specific psychological interventions are typically not indicated for patients with low disability levels. Additionally, the HIT-6 scores in this study align with those reported in RCTs investigating psychological therapeutic interventions on migraine [58]. Furthermore, the operationalization of chronic migraine in this study may have led to an underestimation, as only the number of days with typical migraine symptoms was recorded to assess attack frequency, differing from the ICHD-3 criteria [2]. A further important limitation is that concomitant primary headache disorders or secondary headaches, as well as information on prophylactic medications or preventive treatment modalities, were not systematically assessed or collected, which may have introduced confounding. For example, frequent use of analgesic medication has been associated with higher ictal disability [59]. Finally, pain intensity, attack frequency and disability were assessed via a retrospective self-report questionnaire covering a four-week period. It should be noted that the use of patient-reported questionnaires versus diaries is a matter of controversy [47, 60, 61].

The study also has strengths. It is based on a theory-driven approach that integrates biological and psychological factors evident in migraine and provides a solid framework for developing a migraine-specific explanatory model of disability. The findings suggest significant implications for both clinical practice and future research.

## Conclusion

In this study, sociodemographic and migraine characteristics, as well as psychological factors, were identified as important factors associated with migraine-related ictal disability. Higher disability was significantly associated with subjective pain experience (maximum pain intensity) and maladaptive cognitive-affective processing (pain catastrophizing), which is in line with the assumptions of the FAM. Attack frequency was not significantly associated with disability. While this finding aligns with previous research suggesting that headache frequency may also be more closely related to interictal disability, other factors (e.g. the high proportion of participants reporting severe disability or the use of self-reported data) may also contribute to this lack of association. Taken together, these findings point to the clinical relevance of pain catastrophizing, particularly in patients with higher levels of disability. Future studies should explore causal pathways and investigate whether reducing pain catastrophizing improves migraine-related outcomes.

## Data Availability

The data of this study will be made available to other researchers upon reasonable request after the completion of the study.

## Appendix

**Table 6:** Results of mediation model 1, examining the relationship between maximum pain intensity and disability, with pain catastrophizing as the mediator

	*R*	*R* ^*2*^	*p*	*ß*	*B*	*SE(HC3)*_*B*_	95 %-CI	
							Lower bounds	Upper bounds
Predictors of PCS	0.65	0.42	**<0.001**	-	-	-	-	-
Intercept	-	-	0.176	-	5.34	3.93	-2.42	13.12
Gender	-	-	0.563	-0.04	-1.20	2.07	-5.30	2.90
Age	-	-	0.781	-0.02	-0.02	0.06	-0.13	0.10
Depression	-	-	**<0.001**	0.53	1.11	0.14	0.83	1.39
Maximum Pain Intensity	-	-	**<0.001**	0.25	1.70	0.41	0.88	2.52
Predictors of HIT-6 with mediation	0.79	0.62	**<0.001**	-	-	-	-	-
Intercept	-	-	**<0.001**	-	46.17	3.12	40.01	52.33
Gender	-	-	**0.018**	-0.14	-2.27	0.95	-4.15	-0.40
Age	-	-	**0.032**	0.11	0.05	0.02	0.00	0.10
Depression	-	-	0.103	0.12	0.13	0.08	-0.03	0.28
Maximum Pain Intensity	-	-	**<0.001**	0.47	1.62	0.35	0.93	2.30
Pain Catastrophizing	-	-	**<0.001**	0.35	0.18	0.04	0.10	0.25
Predictors of HIT-6 without mediation (total effect)	0.74	0.55	**<0.001**	-	-	-	-	-
Intercept	-	-	**<0.001**	-	47.13	3.30	40.61	53.64
Maximum Pain Intensity	-	-	**<0.001**	0.56	1.92	0.39	1.15	2.69
Age	-	-	**0.054**	0.11	0.05	0.03	-0.00	0.10
Gender	-	-	**0.016**	-0.15	-2.49	1.02	-4.51	-0.47
Depression	-	-	**<0.001**	0.30	0.33	0.07	0.19	0.46

Significant results in bold

*SE(HC3)* robust standard error, *CI* Confidence interval, *EM* episodic migraine

**Table 7:** Results of mediation model 2, examining the relationship between attack frequency and disability, with pain catastrophizing as the mediator

	*R*	*R* ^*2*^	*p*	*ß*	*B*	*SE(HC3)*_*B*_	95 %-CI	
							Lower bounds	Upper bounds
Predictors of PCS	0.61	0.37	**<0.001**	-	-	-	-	-
Intercept	-	-	**<0.001**	-	18.23	2.96	12.38	24.08
Gender	-	-	0.172	-0.10	-3.24	2.36	-7.90	1.43
Age	-	-	0.729	-0.02	-0.02	0.06	-0.14	0.10
Depression	-	-	**<0.001**	0.61	1.28	0.14	1.00	1.56
Attack Frequency	-	-	0.257	-0.07	-0.14	0.13	-0.39	0.11
Predictors of HIT-6 with mediation	0.67	0.45	**<0.001**	-	-	-	-	-
Intercept	-	-	**<0.001**	-	56.56	2.07	52.47	60.65
Gender	-	-	**0.014**	-0.23	-3.87	1.56	-6.95	-0.80
Age	-	-	0.110	0.11	0.05	0.03	-0.01	0.11
Depression	-	-	**0.043**	0.16	0.17	0.08	0.01	0.34
Attack Frequency	-	-	0.442	-0.05	-0.06	0.08	-0.21	0.09
Pain Catastrophizing	-	-	**<0.001**	0.51	0.26	0.05	0.16	0.36
Predictors of HIT-6 without mediation (total effect)	0.53	0.28	**<0.001**	-	-	-	-	-
Intercept	-	-	**<0.001**	-	61.29	1.98	57.38	65.20
Attack Frequency	-	-	0.232	-0.09	-0.10	0.08	-0.26	0.06
Age	-	-	0.193	0.10	0.05	0.03	-0.02	0.11
Gender	-	-	**0.014**	-0.29	-4.71	1.89	-8.45	-0.98
Depression	-	-	**<0.001**	0.47	0.50	0.07	0.36	0.65

Significant results in bold

*SE(HC3)* robust standard error, *CI* Confidence interval, *EM* episodic migraine

**Table 8:** Results of exploratory mediation model 3, examining the relationship between pain catastrophizing and disability, with fear of attacks as the mediator

	*R*	*R* ^*2*^	*p*	*ß*	*B*	*SE(HC3)*_*B*_	95 %-CI	
							Lower bounds	Upper bounds
Predictors of FAMI	0.67	0.46	**< 0.001**	-	-	-	-	-
Intercept	-	-	**< 0.001**	-	57.02	6.09	44.99	69.06
Age	-	-	0.134	0.18	0.10	0.12	-0.06	0.42
Gender	-	-	0.112	-6.64	-0.11	4.16	-14.86	1.58
Depression	-	-	**0.006**	0.77	0.19	0.28	0.22	1.33
Pain Catastrophizing	-	-	**< 0.001**	1.03	0.54	0.15	0.73	1.32
Predictors of HIT-6 with mediation	0.67	0.45	**< 0.001**	-	-	-	-	-
Intercept	-	-	**< 0.001**	-	54.51	3.08	48.42	60.59
Age	-	-	0.109	0.05	0.10	0.03	-0.01	0.10
Gender	-	-	**0.021**	-3.70	-0.23	1.59	-6.83	-0.57
Depression	-	-	0.112	0.13	0.12	0.08	-0.03	0.30
Pain Catastrophizing	-	-	**< 0.001**	0.23	0.46	0.06	0.10	0.36
FAMI	-	-	0.346	0.03	0.11	0.03	-0.03	0.09
Predictors of HIT-6 without mediation (total effect)	0.67	0.45	**< 0.001**	-	-	-	-	-
Intercept	-	-	**< 0.001**	-	56.12	1.97	52.24	60.01
Age	-	-	0.099	0.05	0.11	0.03	-0.01	0.11
Gender	-	-	**0.008**	-3.89	-0.24	1.46	-6.78	-1.00
Depression	-	-	0.796	0.16	0.15	0.09	-0.19	0.33
Pain Catastrophizing	-	-	**< ** **0.001**	0.26	0.51	0.53	0.16	0.37

Significant results in bold

*SE(HC3)* robust standard error, *CI* Confidence interval, *EM* episodic migraine

## Abbreviations

FAM: Fear Avoidance Model
EM: Episodic migraine
CM: Chronic migraine
